# Functional, Cohort-Level Assessment of CFTR Modulator Responses Using Biobanked Nasal Epithelial Cells from Individuals with Cystic Fibrosis

**DOI:** 10.3390/jpm16010051

**Published:** 2026-01-15

**Authors:** Bente L. Aalbers, Gimano D. Amatngalim, Ellen M. Aarts, Lisa W. Rodenburg, Loes A. den Hertog-Oosterhoff, Harry G. M. Heijerman, Jeffrey M. Beekman

**Affiliations:** 1 Department of Pulmonology, University Medical Center Utrecht, 3584 EA Utrecht, The Netherlandsh.g.m.heijerman@umcutrecht.nl (H.G.M.H.); 2 Lab of Cellular Disease Models, Department of Pediatrics, Regenerative Medicine Center Utrecht, University Medical Center Utrecht, 3584 CT Utrecht, The Netherlands; 3 Centre for Living Technologies, Alliance Eindhoven University of Technology, Wageningen University & Research, University Medical Center Utrecht, Utrecht University, 3584 CB Utrecht, The Netherlands

**Keywords:** cystic fibrosis, nasal epithelial cells, air-liquid interface (ALI) cultures, Ussing chamber, theratyping, biobanking

## Abstract

**Background/Objectives**: Individual responses to CFTR modulators vary widely among people with cystic fibrosis (pwCF), underscoring the need for functional approaches that provide biological context alongside genotype-based therapy selection. Nasal epithelial cultures provide an individual-specific model for theratyping, but most studies rely on freshly isolated cells, restricting repeated testing and long-term sample use. In this study, we tested whether CFTR modulator responses measured in biobanked nasal cells were associated with real-world clinical outcomes. **Methods**: Cryopreserved nasal epithelial cells from 23 pwCF were differentiated at the air–liquid interface and assessed for CFTR modulator-responsive ion transport using Ussing chambers. *In vitro* responses were correlated with 6-month changes in sweat chloride concentration (SCC), FEV_1_, and BMI. **Results**: Cryopreserved cultures retained donor-specific CFTR modulator responsiveness. Modulator-induced forskolin/IBMX-stimulated currents correlated with changes in SCC (R = −0.512). CFTR inhibitor-sensitive currents correlated with FEV_1_ (R = 0.564). Associations between forskolin/IBMX-stimulated currents and FEV_1_ were positive but did not reach statistical significance using two-tailed analysis. BMI changes showed no significant association. **Conclusions**: Biobanked nasal epithelial cultures preserve clinically relevant CFTR modulator responses at the cohort level, supporting their use as functional assays for population-level assessment in cystic fibrosis. This cryopreservation-based strategy enables repeated testing and may expand access to theratyping beyond freshly obtained samples.

## 1. Introduction

Cystic fibrosis (CF) is caused by pathogenic variants in the cystic fibrosis transmembrane conductance regulator (CFTR) gene, leading to impaired airway epithelial chloride and bicarbonate transport, chronic respiratory infection, and progressive lung function decline [1]. The introduction of CFTR modulator therapies has fundamentally changed CF care by directly targeting the underlying molecular defect. However, despite their transformative impact, clinical responses to CFTR modulators vary widely among people with CF (pwCF), including among individuals sharing the same CFTR genotype [2,3]. This marked inter-individual variability has stimulated growing interest in functional approaches that assess CFTR modulator responsiveness directly in patient-derived epithelial cells, thereby providing biological context beyond genotype alone [4].

One widely used functional assay is the measurement of CFTR-mediated ion transport using Ussing chambers in air–liquid interface-differentiated human nasal epithelial cells (ALI-HNEC) obtained from minimally invasive nasal brushings [5]. Compared with conducting airway epithelial cells, HNEC can be sampled repeatedly with low burden to the patient, making them particularly suitable for functional testing in a clinical and research setting. Importantly, comparative studies have demonstrated that CFTR modulator responses measured in nasal and bronchial airway epithelial cultures are highly concordant in Ussing chamber assays, supporting the use of HNEC as a biologically relevant surrogate for bronchial epithelial cells [6]. Previous studies have shown that Ussing chamber measurements in freshly obtained ALI-HNEC can associate *in vitro* CFTR modulator responses with treatment efficacy in pwCF carrying rare CFTR variants [7,8,9]. In addition, several cohort-based studies using nasal epithelial cells have demonstrated significant correlations between *in vitro* CFTR function and clinical outcomes, including reductions in sweat chloride concentration and improvements in lung function following CFTR modulator therapy [10,11,12,13,14]. Together, these findings support HNEC-based theratyping as a complementary functional strategy that can contextualize genotype-based treatment selection at the population level.

Despite this progress, most functional theratyping studies rely on freshly obtained nasal epithelial cells. This dependence imposes practical limitations, including the need for repeated nasal brushings, restricted sample availability, and limited feasibility for longitudinal studies or retrospective testing of emerging CFTR modulators. Cryopreservation of HNEC offers a potential solution by enabling the establishment of epithelial biobanks that preserve donor-specific material for repeated, standardized, or delayed functional analyses [15,16,17]. However, while cryopreserved HNEC have been shown to retain epithelial identity and electrophysiological properties, it remains insufficiently established whether CFTR modulator responses measured after cryostorage preserve clinically relevant cohort-level associations with real-world treatment outcomes.

In previous work, we developed chemically defined, feeder-free protocols for the expansion and differentiation of nasal brushing–derived HNEC and demonstrated preservation of epithelial phenotype and CFTR function following cryopreservation [15,16]. Building on these methodological advances, the present study aimed to determine whether CFTR modulator responses measured in cryopreserved ALI-HNEC from pwCF associate with clinical changes in sweat chloride concentration, lung function, and body mass index following treatment initiation. Specifically, our aim was to determine whether previously reported group-level functional associations are preserved in biobanked epithelial cells. By addressing this question, we provide proof-of-concept for the use of cryopreserved HNEC as a scalable platform for functional assessment in CF.

## 2. Materials and Methods

### 2.1. Cell Culture of Nasal-Brushing-Derived Epithelial Cells

Nasal brushings of pwCF (n = 23) and a non-CF control subject were collected and biobanked between 2018 and 2020, with informed consent, under approval from the TcBIO biobank ethics committee, University Medical Center Utrecht (sub-biobank: 16-586, 25 January 2017 of approval). The non-CF control sample was used as a technical control of the Ussing chamber measurements. Use of biobanked nasal epithelial cells for experimental research, including retrospective analyses, was approved by the TcBIO biobank ethics committee, University Medical Center Utrecht, on 29 May 2020 (release protocol ID: 19/720). Human nasal epithelial cells (HNEC) were isolated, cryopreserved, and further cultured as previously described in our protocol paper [16]. For CFTR function measurements, HNEC cryostored at passage 2 (P2) were thawed and further expanded in one well of a 6-well culture plate pre-coated with 50 μg/mL Collagen IV (Sigma-Aldrich, St. Louis, MO, USA, Cat#C7521) under feeder-free, chemically defined conditions using an expansion medium described in Table S1. Cells we used at passage 4 (P4) post-thaw for differentiation at the air–liquid interface (ALI), as previously described in our protocol paper [16]. In brief, 6.5-mm Transwell inserts (0.4 μm polyester membrane, Corning, Corning, NY, USA, Cat#3470) pre-coated with 30 μg/mL PureCol Type I Collagen Solution (Advanced BioMatrix, Carlsbad, CA, USA, Cat#5005) were seeded with 2 × 10^5^ cells and differentiated in ALI-differentiation medium described in Table S2 for 18 days. Differentiation was monitored by transepithelial electrical resistance (TEER) measurements (EVOM2, World Precision Instruments, Sarasota, FL, USA). 

### 2.2. Quantitative Real-Time PCR (qPCR)

Total RNA was isolated from ALI-HNEC using the RNeasy Mini Kit (Qiagen, Venlo, The Netherlands, Cat#74104), and complementary DNA (cDNA) was synthesized using the iScript cDNA synthesis kit (Bio-Rad, Hercules, CA, USA, Cat#1708891), according to the manufacturer’s instructions. Quantitative real-time PCR (qPCR) was performed to assess the expression of airway epithelial marker genes for basal cells (*TP63*, *KRT5*), secretory cells (*SPDEF*, *AGR2*, *MUC5AC, SCGB1A1*), and ciliated cells (*FOXJ1*, *DNAI1)* using gene-specific primers listed in Table S3. qPCR reactions were carried out using iQ SYBR Green Supermix (Bio-Rad, Cat#1708880) on a CFX96 real-time detection system (Bio-Rad). Relative gene expression was calculated using the ΔCt method and normalized to the housekeeping genes *ATP5B* and *RPL13A* using CFX Manager 3.1 software (Bio-Rad) and Excel (Microsoft, Redmond, WA, USA).

### 2.3. Immunofluorescence Staining and Imaging

Undifferentiated HNEC cultured on collagen IV-coated 96-well plates (Greiner Bio-One, Alphen aan den Rijn, The Netherlands, Cat#655182) and ALI-HNEC were processed for immunofluorescence (IF) staining as previously described [15], with minor modifications. Briefly, cultures were fixed in 4% (*w*/*v*) paraformaldehyde in phosphate-buffered saline (PBS) for 15 min at room temperature, followed by permeabilization in 0.25% (*v*/*v*) Triton X-100 in PBS for 30 min. Non-specific binding was blocked by incubation in blocking buffer consisting of 1% (*w*/*v*) bovine serum albumin (BSA) and 0.25% (*v*/*v*) Triton X-100 in PBS for 60 min. Primary antibodies (listed in Table S4) were diluted in blocking buffer and applied to the cultures at a dilution of 1:500, followed by incubation for 1–2 h at room temperature or overnight at 4 °C, depending on the antibody. After primary antibody incubation, samples were washed three times with PBS and incubated with fluorophore-conjugated secondary antibodies (1:500), in combination with phalloidin, for 30 min in the dark. Following three additional PBS washes, Transwell membranes were excised from the inserts and mounted on glass slides. Both undifferentiated HNEC and ALI-HNEC were mounted using ProLong Gold Antifade Mountant (Thermo Fischer Scientific, Waltham, MA, USA), with DAPI for nuclear counterstaining. Fluorescence images were acquired using a Leica THUNDER imager or a Leica SP8X confocal microscope (Leica Microsystems, Wetzlar, Germany), using identical acquisition settings within experiments. Image processing and visualization were performed using Leica Application Suite X (LAS X), version 5.3.0 (Leica Microsystems).

### 2.4. Ussing Chamber Measurements

Short-circuit current (I_sc_) measurements were performed on fully differentiated ALI-HNEC cultures using a voltage-clamp Ussing chamber system (Physiologic Instruments, Venice, FL, USA). Prior to measurements, Transwell inserts were equilibrated for 20–30 min under open-circuit conditions at 37 °C in bicarbonate-buffered solutions. Measurements were conducted under an apical–basolateral chloride gradient by using asymmetric buffer compositions, with apical and basolateral solutions as detailed in Table S5. Bath solutions were continuously gassed with 95% O_2_/5% CO_2_ to maintain physiological pH and temperature. ALI-HNEC cultures from F508del/F508del donors were pretreated for 48 h with VX-809 (10 µM; Selleck Chemicals, Cologne, Germany, Cat#S1565), VX-661 (10 µM; Selleck Chemicals, Cat#S7059), and VX-445 (5 µM; MedChemExpress, Sollentuna, Sweden, Cat#HY-11177), or vehicle (DMSO). After mounting, cultures were voltage-clamped to 0 mV, and I_sc_ was continuously recorded. To measure CFTR-mediated currents, ALI-HNEC were sequentially stimulated with Benzamil (5 µM, apical; Sigma-Aldrich, Cat#B2417) to block epithelial sodium channel (ENaC)-mediated currents, VX-770 to potentiate CFTR activity (5 µM, apical/basolateral; Selleck Chemicals, Cat#S1144), forskolin (10 µM; Sigma-Aldrich, Cat#F3917) and IBMX (100 µM; Sigma-Aldrich, Cat#I5879) (apical and basolateral) to maximally activate CFTR via cAMP signaling, and CFTR Inhibitor-172 (CFTRInh-172) (5 µM, apical; Sigma-Aldrich, Cat#219670) to specifically inhibit CFTR and confirm current specificity. Tracings were recorded using PowerLab/LabChart 6 software (AD Instruments, Oxford, UK). CFTR function was quantified as either the change in current following forskolin/IBMX stimulation relative to baseline (ΔI_sc_ FSK/IBMX) or as the CFTR inhibitor–sensitive current, defined as the decrease in Isc following CFTRInh-172 relative to the preceding forskolin/IBMX-stimulated plateau (ΔI_sc_ CFTRi).

### 2.5. Data Analysis

Ussing chamber measurements were performed on independent donors, with two technical replicates per experimental condition for each donor. For each Transwell, CFTR-dependent current responses were quantified as described above and averaged across technical replicates. Graphs were made with GraphPad Prism 8 (GraphPad Software Inc., Boston, MA, USA). Statistical analysis was conducted using IBM SPSS 25.0 and GraphPad Prism 8. Associations between *in vitro* CFTR functional measurements and clinical outcome parameters, including changes in sweat chloride concentration (SCC), lung function (FEV_1_% predicted), and body mass index (BMI) over 6 months of treatment, were assessed using Spearman’s rank correlation coefficient. This nonparametric approach was selected because it does not assume normal data distribution and is less sensitive to outliers, which is appropriate for analyses involving small and clinically heterogeneous cohorts. All correlation analyses were performed using two-tailed tests, and statistical significance was defined as *p* < 0.05.

## 3. Results

### 3.1. Cohort Characteristics

A total of 23 pwCF were included in this study, comprising 12 males and 11 females, with an age range of 6 to 43 years at the time of study inclusion. All participants started CFTR modulator therapy as part of routine clinical care and had available baseline and 6-month follow-up clinical data. The distribution of CFTR genotypes and corresponding modulator therapies is summarized in Table 1. The largest subgroup consisted of individuals homozygous for F508del (n = 12), with the majority treated with lumacaftor/ivacaftor (n = 10), while a smaller subset received elexacaftor/tezacaftor/ivacaftor (ETI) (n = 2). Seven participants carried the F508del/S1251N genotype and were treated with ivacaftor monotherapy, reflecting established genotype–drug eligibility. Additional genotypes included F508del/G1249R, F508del/R117H (7T), F508del/Y1092X, and F508del/c.3717+5G>T, each represented by a single individual and treated according to current clinical guidelines. Overall, ivacaftor was used in 9 participants, lumacaftor/ivacaftor in 10 participants, and ETI in 4 participants. Baseline clinical characteristics and 6-month changes in FEV_1_% predicted, SCC, and BMI are summarized by treatment group in Table 2, with per-subject data provided in Table S6. At baseline, individuals treated with ivacaftor had relatively preserved lung function (mean FEV_1_ 86.3 ± 25.5% predicted), whereas those receiving lumacaftor/ivacaftor and ETI had lower baseline FEV_1_ values (61.0 ± 23.1% and 31.3 ± 2.6%, respectively), reflecting differences in disease severity and treatment indication. Across all treatment groups, CFTR modulator initiation was associated with reductions in SCC at 6 months. The largest mean reduction was observed in the ivacaftor group (−53.3 ± 21.0 mmol/L), followed by the ETI group (−47.5 ± 7.3 mmol/L) and the lumacaftor/ivacaftor group (−21.3 ± 8.9 mmol/L). Improvements in lung function were also observed across groups, with mean increases in FEV_1_ of +3.3 ± 6.0%, +5.2 ± 6.7%, and +16.0 ± 10.7% predicted in the ivacaftor, lumacaftor/ivacaftor, and ETI groups, respectively. In addition, modest increases in BMI were observed in all treatment groups.

### 3.2. Characterization of Cryopreserved HNEC Cultures

**Figure 1:**
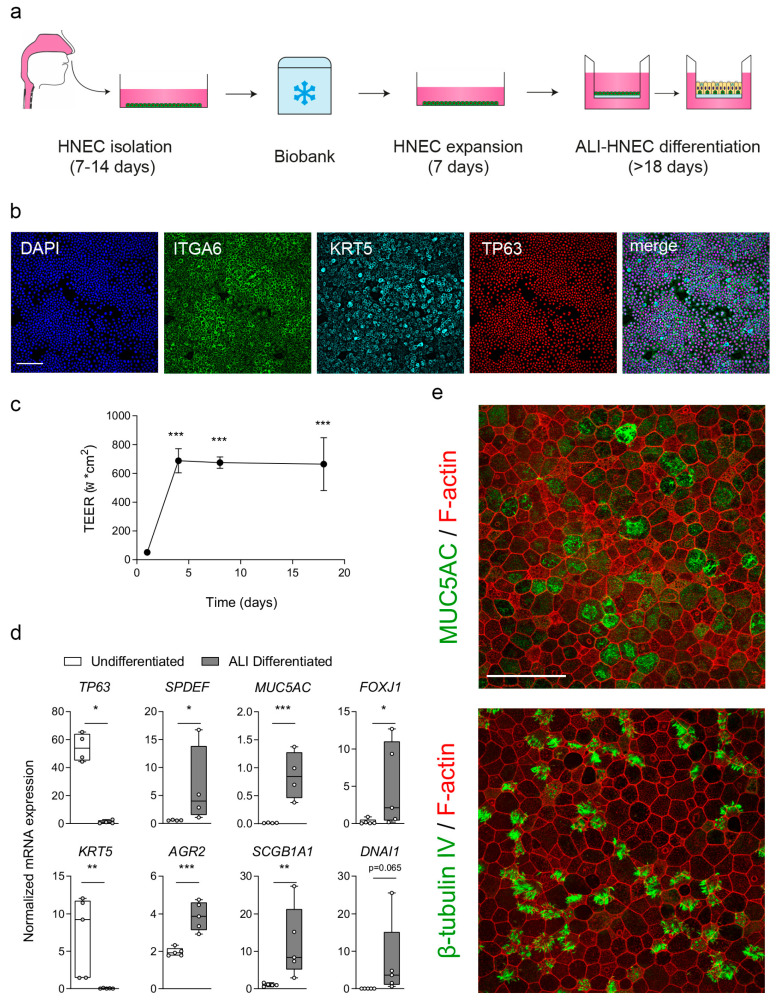
Characterization of cryopreserved nasal epithelial cell cultures. (**a**) Workflow for isolation, cryopreservation, and ALI differentiation of HNEC. (**b**) Immunofluorescent staining of undifferentiated HNEC showing nuclei (DAPI, blue), ITGA6 (green), KRT5 (cyan), and p63 (red). Scale bar = 100 μm. (**c**) Transepithelial electrical resistance (TEER) during ALI differentiation (n = 3 independent donors). Data are means ± SD. TEER values are shown as Ω × cm^2^ (means ± SD). (**d**) Gene expression of basal (*TP63*, *KRT5*), secretory (*SPDEF*, *AGR2*, *MUC5AC*, *SCGB1A1*), and ciliated (*FOXJ1*, *DNAI1*) markers in undifferentiated versus ALI-differentiated HNEC (n = 4–5 donors). Expression normalized to *ATP5B* and *RPL13A*, and is shown as boxplots with whiskers from minimum to maximum. (**e**) Confocal imaging of day-18 ALI-HNEC showing MUC5AC^+^ secretory cells (green) and β-tubulin IV^+^ ciliated cells (green), with phalloidin (red). Scale bar = 50 μm. Analysis of difference was conducted with a one-way ANOVA with Bonferroni post hoc test (**c**), and paired *t*-test (**d**). * *p* < 0.05, ** *p* < 0.01, and *** *p* < 0.001.

To assess whether cryopreserved cells retained basal progenitor identity and differentiation capacity, HNEC cultures were characterized in a subset of donors selected from the overall cohort. Nasal epithelial cells from pwCF were isolated from nasal brushings, expanded to passage 2 (P2), cryopreserved, subsequently thawed, and further expanded before differentiation at air–liquid interface (ALI) at passage 4 (P4) (Figure 1a). Following thawing and expansion, undifferentiated HNEC displayed robust immunofluorescent (IF) staining for integrin α6 (ITGA6), cytokeratin 5 (KRT5), and p63, indicating an airway basal stem/progenitor cell phenotype (Figure 1b). Upon initiation of ALI differentiation, HNEC formed an intact epithelial barrier, as shown by increasing TEER values during the first week of ALI and persistence of barrier function throughout 18 days (Figure 1c). Compared to undifferentiated cultures, ALI-differentiated HNEC showed significantly reduced mRNA expression of basal cell markers and increased expression of secretory and ciliated cell markers, as assessed by qPCR (Figure 1d). Furthermore, differentiated ALI-HNEC contained distinct populations of MUC5AC^+^ secretory cells and β-tubulin IV^+^ ciliated cells, as demonstrated by IF staining (Figure 1e). Together, these data demonstrate that cryopreserved HNEC retain basal progenitor characteristics, form a stable epithelial barrier upon ALI differentiation, and differentiate into mucociliary airway epithelial cells.

### 3.3. CFTR Function and Modulator Responses in ALI-HNEC

Next, CFTR-mediated ion transport was assessed using Ussing chamber measurements in ALI-differentiated HNEC derived from one non-CF control donor and 23 pwCF.

**Figure 2:**
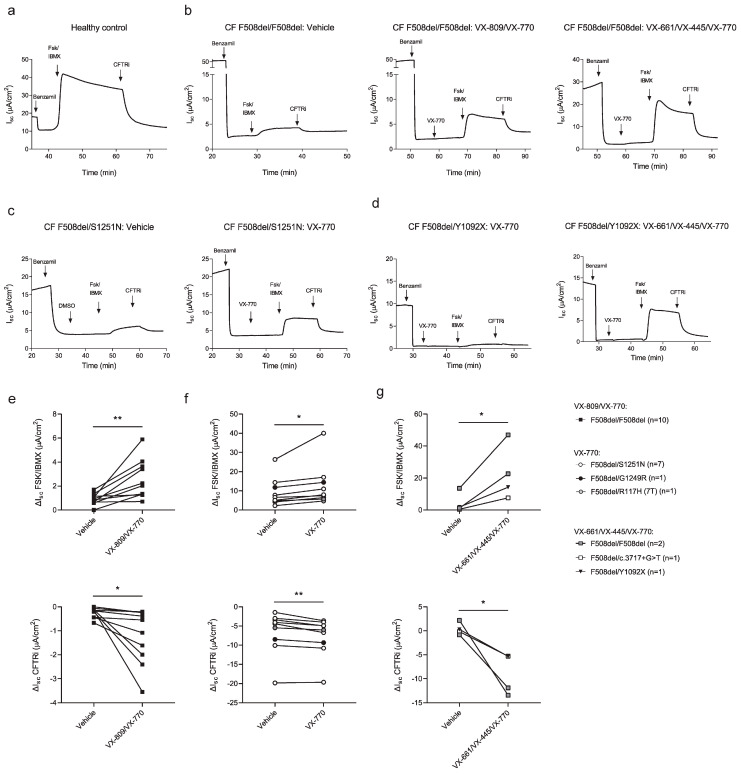
Ussing chamber measurements in ALI-HNEC cultures. (**a**–**d**) Representative short-circuit current tracings from (**a**) non-CF control and pwCF with (**b**) F508del/F508del, (**c**) F508del/S1251N, or (**d**) F508del/Y1092X genotypes. Sequential additions: benzamil, forskolin/IBMX (Fsk/IBMX), VX-770, and CFTRinh-172 (CFTRi). For indicated donors, ALI-HNEC cultures were pre-treated with VX-809, VX-661/VX-445, or vehicle for 48 h. (**e**) Quantification of ΔI_sc_ Fsk/IBMX and ΔI_sc_ CFTRi in F508del/F508del cultures treated with vehicle or VX-809/VX-770 (n = 10). (**f**) Responses to VX-770 in cultures from pwCF with F508del/S1251N (n = 7), F508del/G1249R (n = 1), and F508del/R117H (7T) (n = 1). (**g**) Responses to VX-661/VX-445/VX-770 in cultures from pwCF with F508del/F508del (n = 2), F508del/Y1092X (n = 1), and F508del/c.3717+5G>T (n = 1). Data shown as mean ± SD. * *p* < 0.05, ** *p* < 0.01, paired *t*-test.

Representative short-circuit current (I_sc_) tracings are shown in Figure 2. In non-CF control cultures, stimulation with forskolin/IBMX induced a robust increase in CFTR-dependent chloride current, which was fully inhibited upon addition of the CFTR-specific inhibitor CFTRinh-172, confirming functional CFTR activity in differentiated control cells (Figure 2a). In contrast, ALI-HNEC cultures derived from pwCF exhibited markedly reduced CFTR-mediated currents under baseline conditions, consistent with impaired CFTR function associated with CFTR gene variants (Figure 2b–d). Pretreatment with genotype-appropriate CFTR modulators partially restored CFTR activity. ALI-HNEC cultures from individuals homozygous for F508del showed increased CFTR function following pretreatment with either the corrector–potentiator combination VX-809/VX-770 (Lumacaftor/Ivacaftor) or the triple-combination therapy VX-661/VX-445/VX-770 (ETI), as shown by enhanced forskolin/IBMX-stimulated currents and subsequent inhibition by CFTRinh-172 (Figure 2b,e,g). In cultures carrying the F508del/S1251N genotype, treatment with the CFTR potentiator VX-770 (Ivacaftor) alone resulted in a prominent increase in CFTR-mediated current, consistent with the gating defect associated with S1251N (Figure 2c,f). Cultures derived from pwCF carrying rare CFTR variants, including F508del/G1249R and F508del/R117H (7T), also demonstrated increased CFTR activity in response to VX-770, indicating functional potentiation of residual CFTR activity (Figure 2f). In addition, ALI-HNEC cultures of pwCF with F508del/Y1092X or F508del/c.3717+5G>T gene variants exhibited detectable CFTR responses following treatment with VX-661/VX-445/VX-770 (Figure 2d,g). Quantitative analysis across the cohort showed that CFTR modulator treatment resulted in a significant increase in forskolin/IBMX-induced currents (ΔI_sc_ FSK/IBMX) compared with vehicle-treated cultures (Figure 2e). Similarly, CFTR inhibitor–sensitive currents (ΔI_sc_ CFTRi) were significantly enhanced following modulator treatment, confirming that the observed increases in current were CFTR dependent (Figure 2f,g). While inter-individual variability was observed, these group-level analyses demonstrate a consistent restoration of CFTR function following genotype-appropriate modulator treatment. Together, these data indicate that cryopreserved HNEC differentiated at ALI retain CFTR dysfunction characteristic of CF and exhibit genotype-consistent functional responses to CFTR modulators, supporting their use for functional assessment of CFTR activity in a biobanked setting.

### 3.4. Correlation Between CFTR Modulator Responses in ALI-HNEC and Clinical Outcomes

**Figure 3:**
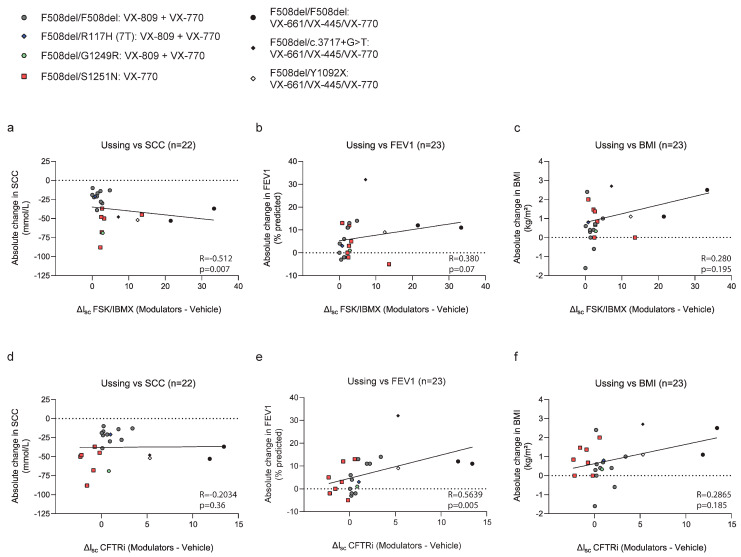
Correlation between Ussing measurements and clinical outcomes. Scatterplots of *in vitro* modulator responses (ΔI_sc_ Fsk/IBMX: (**a**–**c**); Δ_Isc_ CFTRi: (**d**–**f**)) versus changes in (**a**,**d**) sweat chloride concentration (SCC), (**b**,**e**) FEV_1_ (% predicted), and (**c**,**f**) BMI after 6 months of therapy. Each point represents an individual pwCF (n = 23). Spearman’s R and *p* values are shown.

To assess whether CFTR modulator responses measured in cryopreserved ALI-HNEC associate with clinical treatment outcomes, we calculated Spearman rank correlations between *in vitro* electrophysiological readouts and changes in clinical parameters over a 6-month treatment period (Figure 3). Correlation analyses were performed at the cohort level and were not intended to predict individual patient outcomes. SCC data were available for 22 of the 23 subjects, as one individual lacked follow-up measurements. A significant inverse correlation was observed between forskolin/IBMX-stimulated CFTR current (Δ_Isc_ FSK/IBMX) and the change in SCC (R = −0.512, two-tailed *p* = 0.007) (Figure 3a), indicating that greater *in vitro* CFTR activation was associated with larger reductions in sweat chloride. In contrast, CFTR inhibitor–sensitive current (ΔI_sc_ CFTRi) did not correlate significantly with SCC changes (R = −0.203, two-tailed *p* = 0.36) (Figure 3d). For lung function, measured as change in percent predicted FEV_1_, ΔI_sc_ FSK/IBMX showed a positive association with clinical improvement; however, this relationship did not reach statistical significance when assessed using a two-tailed test (R = 0.380, two-tailed *p* = 0.07) (Figure 3b). In contrast, ΔI_sc_ CFTRi demonstrated a significant positive correlation with changes in FEV_1_ (R = 0.564, two-tailed *p* = 0.005) (Figure 3e), suggesting that inhibitor-sensitive CFTR currents may better capture functional aspects of CFTR activity relevant to lung function improvement at the cohort level. No significant correlations were observed between either electrophysiological parameter (ΔI_sc_ FSK/IBMX or ΔI_sc_ CFTRi) and changes in BMI (Figure 3c,f), indicating that CFTR functional responses in ALI-HNEC were not associated with short-term nutritional outcomes in this cohort. Collectively, these results demonstrate that *in vitro* CFTR functional readouts derived from cryopreserved ALI-HNEC associate with clinical response measures at the population level, with distinct electrophysiological parameters showing differential relationships with sweat chloride and lung function outcomes.

## 4. Discussion

This exploratory study examined the correlation between CFTR modulator responses in Ussing chamber measurements of ALI cultures derived from biobanked HNEC and treatment outcomes in pwCF. We observed that modulator-induced increases in CFTR-mediated ion transport were significantly associated with reductions in SCC, and that CFTR inhibitor–sensitive currents were significantly associated with changes in FEV_1_. Forskolin/IBMX-stimulated CFTR currents showed a positive association with changes in FEV_1_, although this did not reach statistical significance. Together, these findings support the presence of physiologically relevant CFTR function in cryopreserved HNEC that is associated with clinical outcomes at the population level. Importantly, these population-level associations do not imply reliable prediction of individual treatment outcomes, but rather reflect group-level functional relationships that are consistent with prior theratyping studies using freshly obtained nasal epithelial cells [10,11,12,13,14,18]. Although correlation strengths were modest, they are comparable to those reported in previous cohort-based studies linking *in vitro* CFTR function to clinical response, supporting the feasibility of cryopreserved cells for functional theratyping at the cohort level.

Besides the usage of cryopreserved HNEC, our study deviates from previous research in several key aspects. Firstly, while previous studies propagated freshly isolated HNEC mainly using feeder cells [10,11,12,13], we followed previously established chemically and growth factor-defined culture conditions for the propagation of HNEC [15,16]. Indeed, Park et al. also examined CFTR modulator responses in HNEC expanded in feeder-free conditions and reported a correlation with changes in SCC [19]. Our findings therefore provide further support for the suitability of feeder-free culture conditions for propagating HNEC used in CFTR modulator response measurements. Secondly, we also employed distinct culture conditions for ALI cultures. This is crucial because a previous comparative study has demonstrated varying effects of ALI-differentiation media on airway epithelial cell differentiation [20], which could result in different responses of CFTR modulators across studies. Furthermore, in contrast to several other studies, we incorporated a chloride gradient during the Ussing measurements. This deliberate inclusion enhances the magnitude of CFTR response to stimuli, as evidenced by previous research conducted by Bratcher and colleagues [21]. While we did not directly compare conditions, our findings add to the evidence that multiple protocols can support clinically relevant readouts.

Limitations of this study should be acknowledged. First, the number of subjects included was modest, and correlations were influenced by variability in a limited and clinically heterogeneous cohort. As in earlier work, clinical outcomes such as FEV_1_ are influenced by multiple factors beyond CFTR function, limiting predictive accuracy at the individual level. Our analyses therefore reflect associative trends at the group level rather than individual-specific predictions. Future studies integrating genetic modifiers and broader clinical data may further enhance functional stratification using biobanked epithelial cultures. In addition, only a single non-CF control was included, which precludes the definition of a normative reference range. We did not perform direct comparisons between cryopreserved and freshly obtained HNEC, nor did we assess inter-experimental reproducibility across multiple freeze–thaw cycles. In the absence of paired fresh-to-cryopreserved comparisons from the same donors, equivalence at the individual-sample level cannot be established. Although prior studies have demonstrated preserved electrophysiological properties after cryopreservation [17], direct within-donor comparisons remain an important next step to confirm functional similarity. Furthermore, all cultures were used at passage 4 post-thaw, and the effect of passage number on CFTR function in this system requires further study. Differences in differentiation and cell-type composition, including variability in ciliated and secretory cells, may also have contributed to heterogeneity in CFTR responses, although this was not systematically quantified in the present study. Finally, follow-up studies are needed to compare this approach with other established theratyping models, such as nasal potential difference and intestinal organoid forskolin-induced swelling assays, which have shown comparable correlations with clinical outcomes [10,18,22].

In summary, this study provides proof-of-concept that Ussing chamber measurements in biobanked nasal cells can detect clinically relevant CFTR modulator responses at the cohort level. This approach may facilitate studies of rare mutations or novel modulators and supports the use of biobanked nasal epithelial cultures for functional precision assessment and population-level stratification. Larger studies, including direct comparisons with freshly isolated cells and other established theratyping models, as well as systematic evaluation of culture conditions, will be required to confirm robustness and support further clinical application.

## Figures and Tables

**Table 1:** CFTR gene variants of included pwCF.

CFTR Gene Variants	n	CFTR Modulator Received *
F508del/F508del	12	Lumacaftor/Ivacaftor (10), ETI (2)
F508del/S1251N	7	Ivacaftor (7)
F508del/G1249R	1	Ivacaftor (1)
F508del/R117H (7T)	1	Ivacaftor (1)
F508del/Y1092X	1	ETI (1)
F508del/c.3717+5G>T	1	ETI (1)
Total	23	Ivacaftor (9), Lumacaftor/Ivacaftor (10), ETI (4)

* Treatment at time of study inclusion. ETI = elexacaftor/tezacaftor/ivacaftor.

**Table 2:** Baseline and 6-month follow-up clinical data of pwCF.

Treatment Group	FEV_1_ (% Predicted)	Sweat Chloride (mmol/L)	BMI (kg/m^2^)
Ivacaftor (n = 9)	86.3 ± 25.5	82.5 ± 19.6	21.4 ± 5.5
	Δ +3.3 ± 6.0	Δ −53.3 ± 21.0	Δ +0.8 ± 0.7
Lumacaftor/Ivacaftor (n = 10)	61.0 ± 23.1	92.5 ± 9.9	21.3 ± 2.1
	Δ +5.2 ± 6.7	Δ −21.3 ± 8.9	Δ +1.7 ± 1.0
ETI (n = 4)	31.3 ± 2.6	93.5 ± 10.9	21.7 ± 2.7
	Δ +16.0 ± 10.7	Δ −47.5 ± 7.3	Δ +1.9 ± 0.9

ETI = elexacaftor/tezacaftor/ivacaftor. Δ = difference after 6-months follow up.

## Data Availability

The original contributions presented in this study are included in the article/Supplementary Materials. Further inquiries can be directed to the corresponding author.

## Supplementary Materials

The following supporting information can be downloaded at https://www.mdpi.com/article/10.3390/jpm16010051/s1: Table S1: Basal cell expansion medium; Table S2: Air-liquid interface (ALI)-differentiation medium; Table S3: qPCR primers; Table S4: Primary antibodies; Table S5: Composition of basolateral and apical buffers used in Ussing chamber assays; Table S6: Per-subject data.

## Abbreviations

The following abbreviations are used in this manuscript:

pwCF	people with cystic fibrosis
CF	Cystic fibrosis
CFTR	Cystic fibrosis transmembrane conductance regulator
ALI	Air-liquid interface
HNEC	Human nasal epithelial cells
BMI	Body mass index
SCC	Sweat chloride concentration
FEV_1_	First second of forced expiration
TEER	Transepithelial electrical resistance
I_sc_	Short-circuit current
ETI	Elexacaftor/tezacaftor/ivacaftor
